# Clinical and immunological response to pneumococcal vaccination in pediatric systemic lupus erythematosus

**DOI:** 10.1186/ar3990

**Published:** 2012-09-27

**Authors:** D Rigdon, AC Gotte, MG Punaro, TB Wright

**Affiliations:** 1 UT Southwestern, Dallas, TX, USA; 2 Children's Medical Center, Dallas, TX, USA

## Background

Sepsis is a leading cause of mortality in pediatric SLE with increased susceptibility to *Streptococcus pneumoniae *infection. We routinely vaccinate with the 23-valent pneumococcal polysaccharide vaccine (PPV) to protect against infection. Response to vaccination may vary due to disease-specific factors and immunosuppressant use. The appropriate time to assess antibody response to PPV is unknown. Our study objectives were to describe the clinical and immunological response to PPV in pediatric SLE and to determine predictors of decline in immunogenicity after PPV.

## Methods

We evaluated 54 pediatric SLE subjects who received the PPV at diagnosis with vaccine titers obtained at varying intervals. Change in disease activity after vaccination was assessed by the SLE Disease Activity Index (SLEDAI). A positive antibody response was defined as ≥7 of the 14 titers measured having a value ≥1 μg/ml. We used Cox proportional hazards to evaluate factors associated with lack of immunological response to PPV.

## Results

The majority of the cohort were female (79%) and 52% were Hispanic ethnicity. Nephritis (74%), cytopenias (57%), and arthritis (50%) were the most common clinical features at baseline presentation. In the month prior to vaccination, 54% of the cohort received pulse methylprednisolone, and 20% received cyclophosphamide or mycophenolate mofetil. There was no change in the median SLEDAI score after vaccination (8 vs. 6, *P *= 0.2). One subject experienced an adverse reaction after initial vaccination, and two subjects developed severe pneumococcal disease despite vaccination. After initial vaccination, 59% of subjects did not achieve protective titers. The median time to inadequate response after initial vaccine was 0.73 years (0.16 to 3.1). In unadjusted models, age (HR = 1.2, *P *= 0.02, 95% CI = 1.02 to 1.4) and hydroxychloroquine use (HR = 2.4, *P *= 0.05, 95% CI = 1.0 to 5.7) were associated with a decreased response. However, after adjustment for age, sex, and ethnicity, disease characteristics and medication use were not associated with lack of immunologic response to PPV.

## Conclusion

PPV was well tolerated in our cohort, but the majority of subjects failed to demonstrate adequate immunologic response to initial vaccination. Disease characteristics and medication use did not explain the lack of response. Pneumococcal infection may occur despite vaccination. PPV vaccination is recommended for patients with pediatric SLE but future studies in larger cohorts are needed to delineate risk factors for lack of immunogenicity.

